# Magnetocaloric Properties of Fe-Ni-Cr Nanoparticles for Active Cooling

**DOI:** 10.1038/srep35156

**Published:** 2016-10-11

**Authors:** V. Chaudhary, R.V. Ramanujan

**Affiliations:** 1 Interdisciplinary Graduate School (IGS), Nanyang Technological University, Singapore 639798, Singapore; 2 Energy Research Institute @ NTU (ERI@N), Nanyang Technological University, Singapore 637553, Singapore; 3 School of Materials Science & Engineering, Nanyang Technological University, Singapore 639798, Singapore

## Abstract

Low cost, earth abundant, rare earth free magnetocaloric nanoparticles have attracted an enormous amount of attention for green, energy efficient, active near room temperature thermal management. Hence, we investigated the magnetocaloric properties of transition metal based (Fe_70_Ni_30_)_100−x_Cr_x_ (x = 1, 3, 5, 6 and 7) nanoparticles. The influence of Cr additions on the Curie temperature (*T*_*C*_) was studied. Only 5% of Cr can reduce the *T*_*C*_ from ~438 K to 258 K. These alloys exhibit broad entropy v/s temperature curves, which is useful to enhance relative cooling power (RCP). For a field change of 5 T, the RCP for (Fe_70_Ni_30_)_99_Cr_1_ nanoparticles was found to be 548 J-kg^−1^. Tunable *T*_*C*_in broad range, good RCP, low cost, high corrosion resistance and earth abundance make these nanoparticles suitable for low-grade waste heat recovery as well as near room temperature active cooling applications.

Energy efficient magnetocaloric materials for magnetic cooling have attracted intense research interest due to unsustainable energy consumption and limitations of current cooling technology^1^,^2^,^3^,^4^,^5^,^6^,^7^,^8^,^9^. A well-known milestone in magnetic cooling is the development of a compressor free wine cooler based on magnetic cooling, developed by Haier, BASF and Astronautics corporation^10^,^11^. Magnetic cooling has already been shown to use 35% less power than conventional cooling^11^. Magnetic cooling is an energy efficient, low noise and low vibration technology which does not use ozone layer depleting hydrofluorocarbons and is, therefore, environmentally friendly^12^. The magnetocaloric effect (MCE) is the change in temperature of a material due to the adiabatic application or removal of an external magnetic field^13^. This temperature change is related to the magnetic entropy change (∆*S*_*M*_). Generally, MCE is large in the vicinity of the Curie temperature (*T*_*C*_), where the magnetic spins undergo an order - disorder phase transition.

Gd_5_(Si_*x*_Ge_1−*x*_)_4_ and other R_5_T_4_ (R = Rare earth, T = Transition metal) materials can exhibit promising magnetocaloric performance and are known as “Giant magnetocaloric materials”^14^,^15^. However, issues surrounding rare-earths are very complex due to strategic reasons and economics. China is the main supplier of rare earths since several decades, accounting for ~97% and ~90% of world production in 2009 and 2013, respectively^16^. This control may result in supply instabilities. In addition, rare earth based materials are corrosion prone and not earth abundant. These undesirable factors motivate us to develop non rare earth based magnetocaloric materials^17^,^18^,^19^,^20^,^21^,^22^,^23^.

First order transition materials (FOTM) which exhibit simultaneous magnetic and structural transition result in high isothermal entropy change^24^,^25^,^26^. However, the narrow working temperature span and large magnetic and thermal hysteresis in FOTM limit real-world applications^5^,^13^,^27^,^28^. The magneto-structural transition is often associated with field and temperature hysteresis, which reduce maximum operating frequency. In addition, the repetitive structural transition in FOTM can cause result in mechanical instability, which cause failure of the system^2^,^29^,^30^. On the other hand, second order transition materials (SOTM) exhibit a magnetic transition. These materials generally have lower isothermal entropy change compared to those of FOTM. However, SOTM are superior in terms of negligible magnetic and temperature hysteresis and also exhibit large working temperature span, and therefore, high relative cooling power (RCP)^5^,^13^,^28^,^31^,^32^. Hence, there is a considerable interest in rare earth free, cost effective and readily available Fe based materials exhibiting a second order magnetic phase transition^5^,^13^,^28^,^31^,^33^,^34^,^35^.

Typically, bulk magnetocaloric materials have been developed for cooling systems. The magnetocaloric effect in nanostructured materials has received considerable interest recently since they possess additional advantages^5^,^13^,^28^,^31^,^32^,^36^,^37^,^38^. These nanomaterials can be useful for active magnetic cooling devices, microfluidic reactors and other systems. Slow heat transfer in bulk solids is one of the most difficult issues which diminish the efficiency of thermal management systems. The dispersion of magnetic particles in a suitable fluid can solve this challenge, the large surface area of nanoparticles and dispersion in fluid results in better thermal contact and therefore faster heat exchange compared to bulk systems. Furthermore, such ferrofluids can be used for self-pumping, automatic, magnetic cooling^28^,^39^,^40^.

γ-Fe_80−x_Ni_x_Cr_20_ (14 ≤ x ≤ 30) alloys have competing exchange interactions, hence the local spin orientation depends on its environment^41^. The effective exchange interaction can be positive, negative, or nearly zero. This exchange interaction is governed by the concentration, distribution, and strength of the six different exchange interactions (*J*_*ij*_) between the different magnetic atoms. By using neutron scattering technique, Men’shikov *et al.*^42^ reported the values of the exchange integrals: *J*_*ij*_ (Ni*–*Ni) = 52 meV, *J*_*ij*_ (Fe*–*Ni) = 36 meV, *J*_*ij*_ (Ni*–*Cr) = 122 meV, *J*_*ij*_ (Fe*–*Cr) = 39 meV, *J*_*ij*_ (Fe*–*Fe) = −7 meV, *J*_*ij*_ (Cr*–*Cr) = −227 meV.

Our earlier studies show that γ-FeNiB nanoparticles are suitable candidates for low grade waste heat recovery while γ-FeNiMn can be used for slightly above room temperature applications^5^,^13^. On the other hand, alloying of iron based material with Cr will improve corrosion resistance^43^, e.g., increasing Cr content in the magnetic Fe_73.5−x_Si_13.5_B_9_Nb_3_Cu_1_Cr_x_ alloy results in a corrosion resistant material for marine or SiO_2_ contaminated environments^44^,^45^. Therefore, Fe-Ni-Cr alloys possess both good corrosion resistance and good magnetocaloric properties.

We report the effect of alloying of Fe_70_Ni_30_ with Cr on magnetic phase transition temperature (*T*_*C*_) and magnetocaloric properties of alloy nanoparticles. Six compositions: Fe_70_Ni_30_, (Fe_70_Ni_30_)_99_Cr_1_, (Fe_70_Ni_30_)_97_Cr_3_, (Fe_70_Ni_30_)_95_Cr_5_, (Fe_70_Ni_30_)_94_Cr_6_, and (Fe_70_Ni_30_)_93_Cr_7_ were synthesized, the nomenclature is Cr0, Cr1, Cr3, Cr5, Cr6 and Cr7, respectively. The theoretical values of *T*_*C*_ were compared with experimental results. In addition, these nanoparticles were coated with oleic acid (80%) and ammonium hydroxide (20%) and dispersed in oleic acid to make the ferrofluid. The ferrofluid was used as a heat transfer medium in a self-pumping magnetic cooling prototype.

## Results

### X-ray Diffraction and Transmission Electron Microscopy Analysis

**Figure 1:**
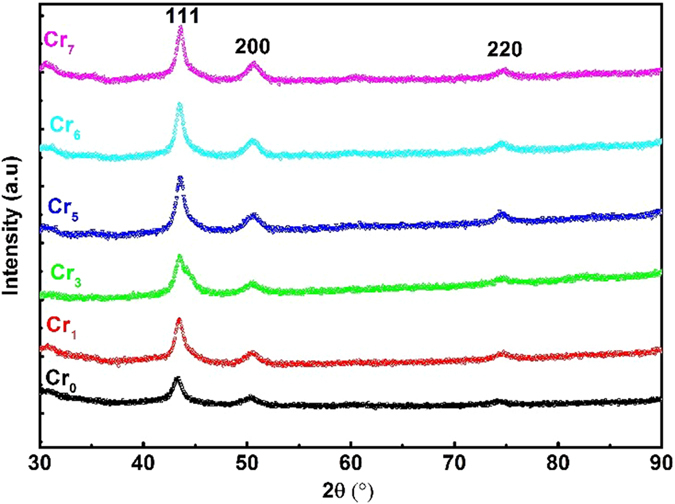
XRD patterns of Cr0, Cr1, Cr3, Cr5, Cr6 and Cr7 nanoparticles after annealing at 700 °C for 2 h and then quenching in water.

Figure 1 shows the XRD patterns of Cr0, Cr1, Cr3, Cr5, Cr6 and Cr7 nanoparticles after heating at 700 °C for 2 h followed by quenching. All the samples exhibit three main diffraction peaks (111, 200 and 220) of the γ-FeNi phase with lattice parameter (a) in the range of 3.5919(4)–3.5983(3) Å and space group Fm-3m. Adding Cr to Fe_70_Ni_30_ does not shift in the diffraction peak positions much as the atomic radius of Cr does not differ much from the corresponding value for Fe and Ni. The average crystal sizes, calculated by the Scherrer formula after subtracting the instrumental line broadening, were ~9 nm, ~12 nm, ~10 nm, ~13 nm, ~12 nm and ~11 nm for Cr0, Cr1, Cr3, Cr5, Cr6 and Cr7 nanoparticles, respectively. All the samples exhibit asymmetric broadening in the 111 diffraction peak.

**Figure 2:**
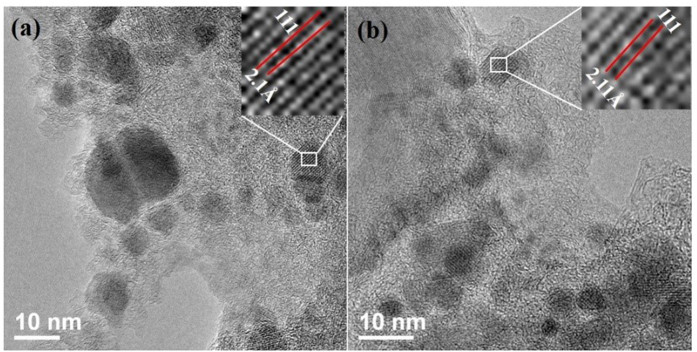
Bright field TEM micrographs of (**a**) Cr3 and (**b**) Cr5 nanoparticles, insets show lattice fringe images corresponding to 111 planes.

Figure 2 shows the bright field transmission electron micrograph of Cr3 and Cr5 nanoparticles. The particle size for Cr3 is in the range of 3 nm to 21 nm, with an average size of 9 nm, while the particle size for Cr5 is in the range of 4 nm to 25 nm range, with an average size of 12 nm. These values are close to the value obtained from XRD data. The lattice fringes of 2.1 Å and 2.11 Å for Cr3 and Cr5, respectively, correspond to the 111 planes of the fcc phase (inset of Fig. 2).

### Curie temperature

**Figure 3:**
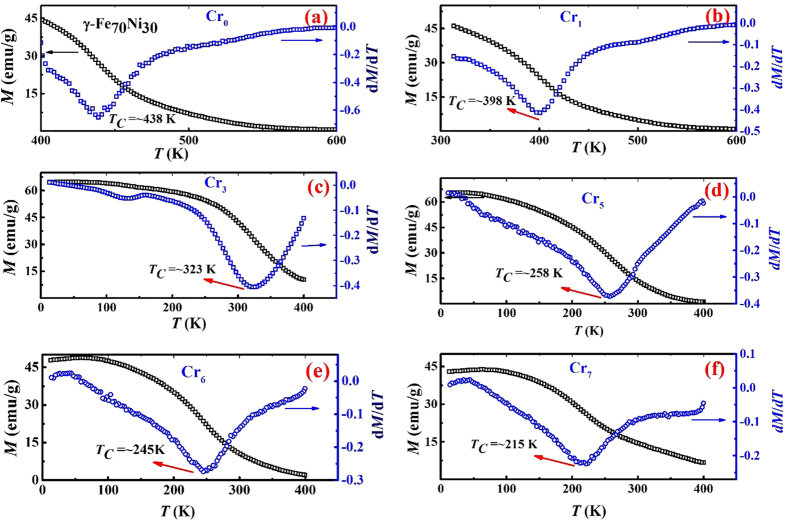
Left axis shows the temperature dependence of magnetization *M*(*T*) for (**a**) Cr0, (**b**) Cr1, (**c**) Cr3, Cr5, Cr6 and Cr7 while the right axis shows the corresponding derivative with respect to temperature (*dM*/*dT*). The Curie temperature for Cr0, Cr1, Cr3, Cr5, Cr6 and Cr7 is 438 K, 398 K, 323 K, 258 K, 245 K and 215 K, respectively.

The Curie temperature is the temperature at which the ferromagnetic phase changes to the paramagnetic phase. For MCE applications, we need to determine the *T*_*C*_ of that material. It should be noted that the MCE is maximum at its *T*_*C*_ and relatively small or almost zero (depending on the *T*_*C*_ distribution and the order of the phase transition) at temperatures away from *T*_*C*_. Fig. 3(a,b,c) shows the temperature dependence of magnetization, *M(T)* (left) and d*M*/d*T* (right) for (Fe_70_Ni_30_)_100−x_Cr_x_ (x = 0, 1, 3, 5, 6 and 7) nanoparticles, measured upon cooling under a field of 0.1 T. The Curie temperatures (*T*_*C*_) of Cr0, Cr1, Cr3, Cr5, Cr6 and Cr7 were found to be 438 K, 398 K, 323 K, 258 K, 245 K and 215 K, respectively.

*T*_*C*_ was determined from the minima of the plot of *dM/dT* versus *T*. The reduction of *T*_*C*_can be understand from the mean field model *T*_*C*_ = *J(r)*_*eff*_ *Z*_*T*_ *S (S* + *1*)/*3k*_*B*_, where *J(r)*_*eff*_ is the effective exchange interaction, *Z*_*T*_ is coordination number, *S* is the atomic spin quantum number and *k*_*B*_ is the Boltzmann constant^5^.

**Figure 4:**
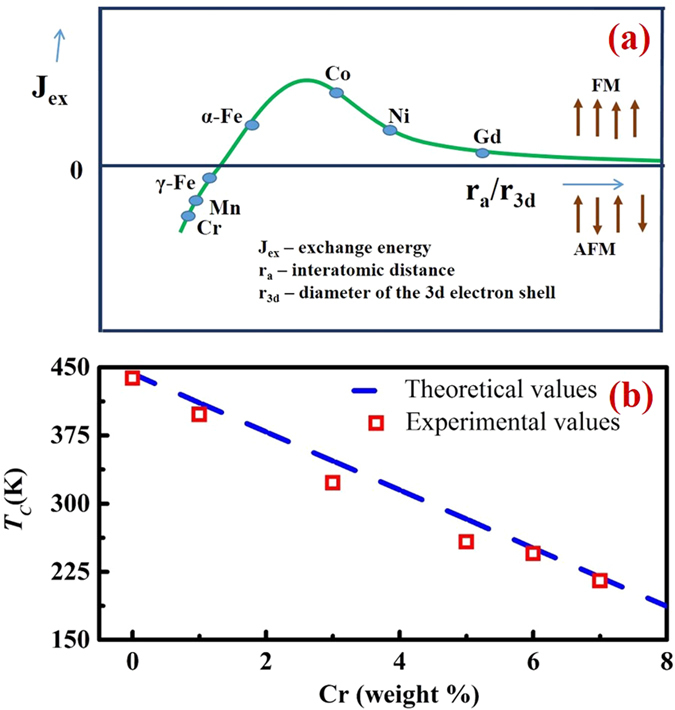
(**a**) The Bethe-Slater curve (schematic) showing the dependence of the exchange interaction on the ratio of interatomic distance to the diameter of the 3d electron shell^46^ (**b**) Phase diagram for the ternary system (Fe_70_Ni_30_)_100−*x*_Cr_x_ for x = 0 to 8. Dashed blue line represents the values predicted from the equation *T*_*C*_ = *T*_*C*1_ + (*T*_*C*_/dc) (**c**) while points (red square) are experimental results.

The Bethe-Slater curve qualitatively describes the variation in strength of direct exchange as a function of the ratio of the interatomic distance to diameter of the 3d electrons (r_a_/r_3d_)^46^. A pair interaction of two atoms sharing two electrons can be used to explain the trend of this curve. A value of 1.5 for ferromagnetic spin coupling was assumed empirically in this curve to separate positive from negative exchange interactions (*Jex*)^46^ (Fig. 4(a)). For a ratio r_a_/r_3d_ less than 1.5, when the electrons from two neighbouring atoms are close to each other, the Pauli Exclusion Principle requires the spins of these electrons to be antiparallel, which results in antiferromagnetic interaction between these atoms. If the ratio r_a_/r_3d_ is greater than 1.5, 3d electrons can be further away from each other, filling two different orbital states, resulting in ferromagnetic interactions. After reaching a maximum value, the exchange coupling starts to decrease because of decreasing spatial overlap of the wave functions of the electrons. For the same value of *x*, *T*_*C*_ for (Fe_70_Ni_30_)_100−*x*_Cr_*x*_is lower than that of (Fe_70_Ni_30_)_100−x_Mn_x_ alloys^5^,^31^. This is because the value of *J*_*CrCr*_is more negative than that of *J*_*MnMn*_. Hence, the effective exchange interaction (*J(r)*_*eff*_) is less in the case of (Fe_70_Ni_30_)_100−x_Cr_x_. The coordination number (*Z*_*T*_) is the same in both cases (due to the same crystal structure), which results in a reduction in *T*_*C*_.

The experimental values of *T*_*C*_ were compared with values calculated from the expression *T*_*C*_ = *T*_*C*1_ + (*dT*_*C*_/*dc*) *c*, *T*_*C*1_ is the Curie temperature of the parent alloy Fe_70_Ni_30_, *dT*_*C*_/*dc* is the rate of change of Curie temperature with concentration (*c*)^46^. The *dT*_*C*_/*dc* value for Cr is −3.2 × 10^3^ K/at %^46^. A value of *T*_*C*_ for Fe_70_Ni_30_ was obtained from the binary Fe-Ni phase diagram. This is close to the experimental value of 438 K. Fig. 4(b) shows the change in Curie temperature with Cr% in the ternary system (Fe_70_Ni_30_)_100−x_Cr_x_.

The dashed blue line and red square represent the expression *T*_*C*_ = *T*_*C*1_ + (*dT*_*C*_/*dc*) *c* and experimental data, respectively. The experimental *T*_*C*_ values for Cr0, Cr1, Cr3, Cr6 and Cr7 are reasonably close to those calculated from the expression. This facile tuning of *T*_*C*_ makes these alloys useful for near room temperature cooling.

### Magnetocaloric Effect

**Figure 5:**
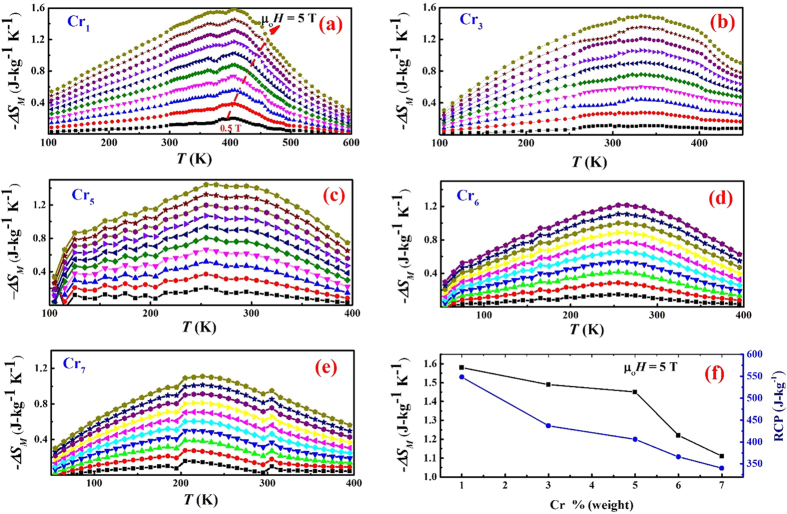
Temperature dependence of magnetic entropy change (−∆S_*M*_) under magnetic field ranging from 0.5 T to 5 T for (**a**) Cr1, (**b**) Cr3, (**c**) Cr5, (**d**) Cr6 and (**e**) Cr7 alloy. (**f**) Dependence of −∆S_*M*_ (left axis, black square) and RCP (right axis, blue circle) on chromium content in (Fe_70_Ni_30_)_100−*x*_Cr_*x*_ nanoparticles at applied magnetic field of 5 T.

Figure 5(a,b,c,d,e) show the temperature dependence of the magnetic entropy change (−∆S_*M*_) under a range of magnetic fields, ranging from 0.5 T to 5 T for Cr1, Cr3, Cr5, Cr6 and Cr7 alloy, respectively. In all cases, the −∆S_*M*_ versus *T* curves are very broad, exhibiting a table-like shape. There are several reports of the desirability of such table-like shape in magnetocaloric materials for real applications^47^,^48^. Comparing our data to the literature, the −∆S_*M*_ and RCP values were calculated at *T*_*C*_. For 1 T applied magnetic field, ∆S_*M*_ for Cr1, Cr3, Cr5, Cr6 and Cr7 at their *T*_*C*_ was found to be 0.38 J-kg^−1^K^−1^, 0.27 J-kg^−1^K^−1^, 0.37 J-kg^−1^K^−1^, 0.29 J-kg^−1^K^−1^ and 0.28 J-kg^−1^K^−1^, respectively. When the field was increased to 5 T, ∆S_*M*_ for Cr1, Cr3, Cr5, Cr6 and Cr7 was found to be 1.58 J-kg^−1^K^−1^, 1.49 J-kg^−1^K^−1^, 1.45 J-kg^−1^K^−1^, 1.22 J-kg^−1^K^−1^ and 1.11 J-kg^−1^K^−1^, respectively.

Figure 5(f) shows the magnetic entropy change (left axis) and RCP (right axis) vs Cr % (weight) in (Fe_70_Ni_30_)_100−*x*_Cr_*x*_ alloy nanoparticles at an applied field of 5 T. Both ∆S_*M*_ and RCP decrease with increasing Cr % in (Fe_70_Ni_30_)_100−*x*_Cr_*x*_, which can be attributed to antiferromagnetic interactions associated with Cr atoms.

**Figure 6:**
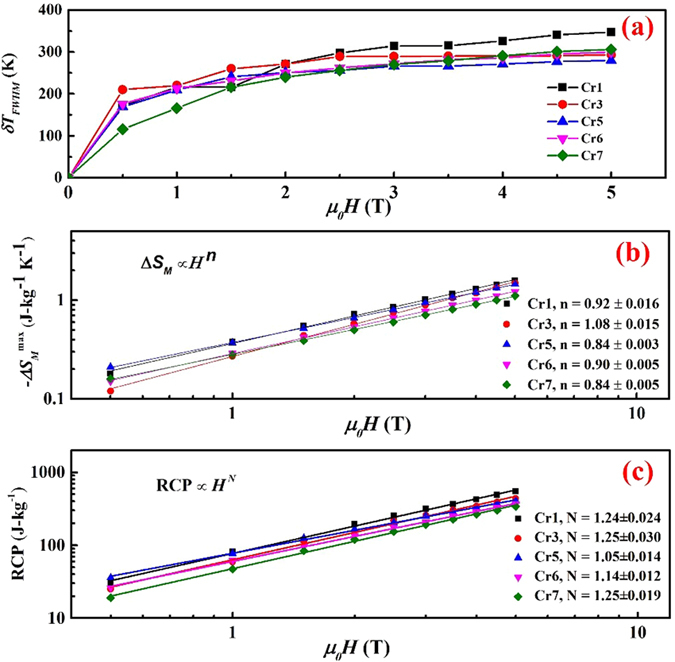
(**a**) Field dependence of working temperature span (δ*T*_*FWHM*_) for Cr1, Cr3, Cr5 Cr6 and Cr7 alloys. (**b**) Variation in relative cooling power (RCP) and (**c**) maximum change in entropy (−∆S_*M*_^max^) as a function of applied field. The plots (**b**) and (**c**) are in log-log scale.

Relative cooling power (RCP) is an important performance metric, it is defined as the product of the maximum change in entropy (∆S_*M*_) and the full width at half maximum (δ*T*_*FWHM*_) of the entropy versus temperature curve, i.e., RCP = ∆S_*M*_ × δ*T*_*FWHM*_. Figure 6(a) shows the variation of δ*T*_*FWHM*_, also known as working temperature span, with applied magnetic field.

The δ*T*_*FWHM*_ for Cr1, Cr3, Cr5, Cr6 and Cr7 was found to be 216 K, 220 K, 209 K, 213 K and 166 K at magnetic field of 1 T, respectively. Our δ*T*_*FWHM*_ values are higher than those of Gd (~35 K)^49^, Pr_2_Fe_17_ (~78 K)^36^, Nd_2_Fe_17_ (~95 K)^36^, (Fe_70_Ni_30_)_89_Zr_7_B_4_ (133 K)^18^ at an applied magnetic field of 1 T. Single and multiphase alloys of (Fe_70_Ni_30_)_89_B_11_ have δ*T*_*FWHM*_ value of 174 K and 322 K, respectively^13^. Our high working temperature span results in high RCP, which quantifies the magnitude of the heat extracted in a thermodynamic cycle. Fig. 6(c) shows the field dependence of RCP on the log-log scale and the corresponding linear fit. The RCP for Cr1, Cr3, Cr5, Cr6 and Cr7 increased from 82 J-kg^−1^, 59 J-kg^−1^, 77 J-kg^−1^, 62 J-kg^−1^ and 47 J-kg^−1^ to 548 J-kg^−1^, 436 J-kg^−1^, 406 J-kg^−1^, 366 J-kg^−1^ and 306 J-kg^−1^ as the field increases from *ΔH* = 1 T to *ΔH *= 5 T, respectively.

From the Arrott-Noakes equation of state, the magnetic entropy change at *T*_*C*_ can be expressed by the relation ∆S_*M*_ α *H*^*n*^, where *n* = 1 + [(β − 1)/(β + γ)]. The field dependence of RCP can be expressed by the power law RCP α *H*^*N*^, with *N* = 1 + 1/δ. β, γ and δ are critical exponents^50^. The linear fit of field dependence of ∆S_*M*_(Fig. 6(b)) and RCP (Fig. 6(c)) at *T*_*C*_ results in values of local exponents “*n*” and “N”. The values of local exponent “*n*” at *T*_*C*_ for Cr1, Cr3, Cr5, Cr6 and Cr7 were 0.92, 1.08, 0.84, 0.90 and 0.84 respectively, and the values of local exponent “N” at *T*_*C*_ for Cr1, Cr3, Cr5, Cr6 and Cr7 were 1.24, 1.25, 1.05, 1.14 and 1.25, respectively. The variation in local exponent can be attributed to different microscopic interactions due to different Cr % in the alloys.

### Ferrofluid based magnetic cooling

**Figure 7:**
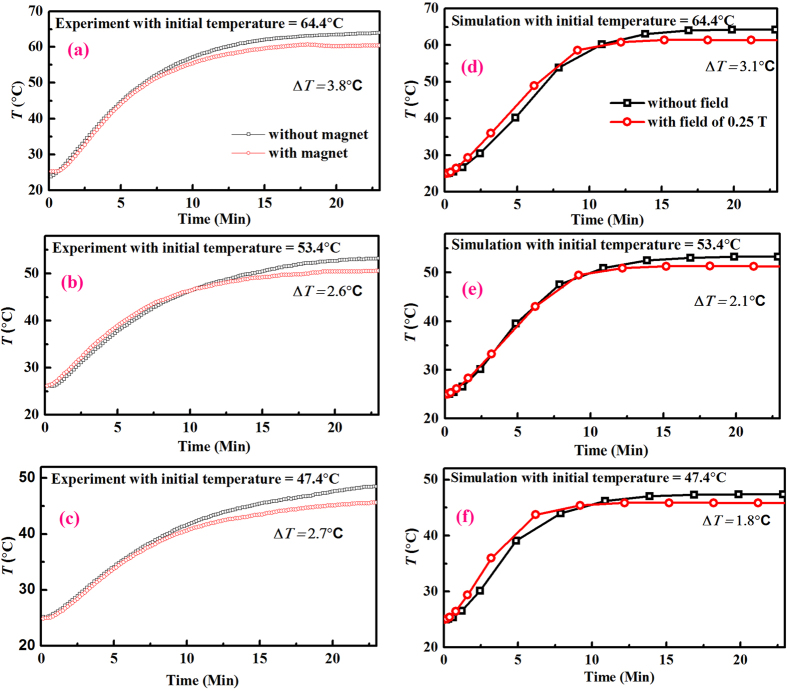
Temperature v/s time for initial temperature of heat load of (**a**) 64.4 °C, (**b**) 53.4 °C and (**c**) 47.4 °C, respectively, without and with magnetic field of 0.25 T. Simulated temperature profiles without and with magnetic field of 0.25 T for corresponding temperature of heat load of (**d**) 64.4 °C, (**e**) 53.4 °C and (**f**) 47.4 °C.

As mentioned in methods, we prepared a ferrofluid based on our nanoparticles which were synthesized by ball milling. To determine the effect of initial temperature of heat load on cooling, the initial heat load temperatures of 64.4 °C, 53.4 °C and 47.4 °C were used. A magnetic field of 0.25 T was applied near the heat load. Figure 7 shows the temperature profiles for heat load for different initial temperature with a magnetic field of 0.25 T and without magnetic field. The results from experiments and simulation show an obvious reduction in temperature (∆*T*) in all cases. The value of ∆*T* increases from 2.7 °C to 3.8 °C when load temperature increases 47.4 °C to 64.4 °C, respectively.

These results show that ferrofluid based magnetic cooling is feasible. Our experimental results were in good agreement with the simulations for the same magnetic field, other parameters are the same as those used in the experiments.

### Discussion

The (Fe_70_Ni_30_)_100−*x*_Cr_x_ (x = 1, 3, 5, 6 and 7) exhibits a second order magnetic phase transition that is tunable from ~438 K to ~215 K. The wide Curie temperature distribution and therefore high RCP, is consistent with the asymmetric nature of 111 diffraction peak in XRD, which implies that the alloys exhibit a range of lattice parameters due to the process of ball milling^51^. This lattice distribution gives high distribution of exchange interaction, which leads to a distribution of *T*_*C*_. The reduction in *T*_*C*_ and ∆S_*M*_ with increasing Cr% is related to the reduction of total exchange energy due to the antiferromagnetic nature of Cr. The Curie temperature and magnetic entropy change of the amorphous alloys Fe_74−x_Cr_*x*_Cu_1_Nb_3_Si_15.5_B_6.5_ (with *x* = 2, 8, 10, 12, 13, 14 and 20) were also found to decrease with increasing Cr content^52^.

Engelbrecht *et al.* reported that for practical cooling systems, a material with a broad peak in entropy change (large δ*T*_*FWHM*_) provides significantly higher cooling power than a material with a sharp peak^53^. The cooling power for a material with low Δ*S*_*M*_ and high δ*T*_*FWHM*_ is greater than that of a material with high Δ*S*_*M*_and low δ*T*_*FWHM*_. Thus, for a magnetic regenerator, a broad temperature distribution of MCE is more attractive than sharp Δ*S*_*M*_ peaks.

One of the main factors for the commercial exploitation of a magnetic material is its cost. Ucar *et al.,* reviewed the RCP in terms of Joule/$ of various magnetocaloric materials and it was found that transition metal based materials have a critical advantage over rare earth based materials^23^. We have estimated the cost of our materials and other relevant magnetocaloric materials. The materials cost of our Fe-Ni-Cr nanoparticles is only about 2% of the cost of pure Gd. Very recently, a transition metal based high entropy alloy NiFeCoCrPd_*X*_ was introduced as a promising magnetocaloric material. The materials cost of our Fe-Ni-Cr is only about 0.3% of the cost of NiFeCoCrPd_0.50._ In addition, our (Fe_70_Ni_30_)_95_Cr_5_ exhibit higher Δ*S*_*M*_ (123%) and RCP (180%) compared to NiFeCoCrPd_0.25_; the *T*_*C*_ is almost the same. Table 1 shows the values of ∆S_*M*_, RCP and cost of our alloys and other magnetocaloric materials.

The RCP values for our alloy nanoparticles are comparable with other key magnetocaloric materials, the better corrosion resistance would enhance their suitability for magnetic fluid applications. As mentioned earlier, nanoparticles exhibit additional advantages compared to the bulk, e.g. they can be dispersed in a suitable liquid and used as a ferrofluid for active cooling^40^. Ferrofluid-based self-pumping has novel applications, e.g., for cooling of microelectronic devices and power electronics system^5^,^54^. Alloy based ferrofluid was used for the first time for magnetic cooling application^55^. The stability of the ferrofluid needs to be improvement for long term application.

## Conclusions

The magnetocaloric properties of Fe-Ni-Cr nanoparticles were studied. Cr was used to tune the *T*_*C*_ of Fe-Ni alloy from more than 400 K to below room temperature. The RCP for Cr1, Cr3, Cr5, Cr6 and Cr7 increased from 82 J-kg^−1^, 59 J-kg^−1^, 77 J-kg^−1^, 62 J-kg^−1^ and 47 J-kg^−1^ to 548 J-kg^−1^, 436 J-kg^−1^, 406 J-kg^−1^, 366 J-kg^−1^ and 306 J-kg^−1^ J-kg^−1^ as field increases from *ΔH* = 1 T to *ΔH* = 5 T, respectively. The cost of our nanoparticles is only ~2% of the cost of pure Gd and Gd_5_Ge_1.9_Si_2_Fe_0.1_ magnetocaloric materials. The magnetocaloric properties, good corrosion resistance and low cost of these nanoparticles makes them attractive for magnetic fluid applications.

## Methods

High energy ball milling is a suitable technique for producing large-scale, nano- and micro sized materials. This technique is based on mechanical energy transfer created by the collision of hard phase materials with the reactants. Mechanical alloying consists of flattening, welding, fracturing and re-welding of the powder by hard grinding balls. Therefore, alloying of nanostructured powders with defined stoichiometry and crystalline order can be achieved. the high energy ball milling of Fe-Ni-Cr alloy particles was performed.

Nanoparticles of (Fe_70_Ni_30_)_100−*x*_Cr_x_ alloy were prepared by high energy planetary ball milling (FRITSCH) at 600 rpm under Ar atmosphere from elemental Fe (99.99%, Sigma Aldrich), Ni (99.998%, Fisher ChemAlert Guide) and Cr (>99%, Sigma Aldrich) powders. The ball to powder ratio was 10:1. The vials and balls were made of zirconium oxide, and the volume of the vial was 125 ml, which contains 15 balls (10 mm in diameter). To prevent oxidation during heat treatment, the magnetic nanoparticles were sealed under high vacuum (10^−5^ torr) in a quartz tube. The sealed tube was heated at 700 °C (γ- phase region) for 2 h and quenched in water^13^. The rate of quenching was ~125 °C/sec. The structure and phase were determined by X-ray diffraction (XRD) using a Bruker D8 Advance diffractometer (CuKα radiation). The composition was confirmed by energy dispersive X-ray spectroscopy using a JEOL JSM-7600F scanning electron microscope. To determine particle size, transmission electron microscopy (TEM) of nanoparticles was carried out on a JEOL 2010 TEM with an operating voltage of 200 kV. Samples for TEM were prepared by ultrasonically dispersing a small amount of powder in hexane, followed by putting a drop of the suspension on a holey carbon-coated copper grid, the sample is then dried overnight in vacuum. The magnetic properties were measured using a physical property measuring system (PPMS) (EverCool-II, Quantum Design), equipped with a vibrating sample magnetometer probe and an oven (model P527). The M (*H*) isotherms with field from 0 to 5 T in steps of 5 K (near *T*_*C*_) and 10 K (elsewhere) were recorded for Δ*S*_*M*_ measurements. The isothermal magnetic entropy change due to application of magnetic field was calculated using a numerical approximation to the Maxwell equation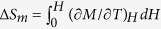, where Δ*S*_*M*_ is the magnetic entropy change, *T* is the temperature, *M* is the magnetization.

These Fe-Ni-Cr nanoparticles were used to prepare the ferrofluid. (Fe_70_Ni_30_)_95_Cr_5_ nanoparticles were functionalized with oleic acid and ammonium hydroxide and subjected high energy ball milling. Subsequently, these coated nanoparticles were dispersed in oleic acid. This ferrofluid of Fe-Ni-Cr nanoparticles and oleic acid was used as the heat transfer medium to perform magnetic cooling.

A 5.2 mm inner diameter, 60 cm circumference polymer tube was used for circular flow. A heat load (electric heater made by Kanthal wires) and a heat sink (cold water) were placed opposite each other. A permanent magnet, which can provide a maximum field of 0.25 T, was placed close to the heat load. A temperature data logger with SD card was used to record temperature v/s time. The initial temperature was tuned by changing current through the Kanthal wire using a Keithley power supply (Model: 2231 A-30-3). For modelling, COMSOL Multiphysics simulation software version 4.4 was used with finite element method and normal mesh.

## Additional Information

**How to cite this article**: Chaudhary, V. and Ramanujan, R.V. Magnetocaloric Properties of Fe-Ni-Cr Nanoparticles for Active Cooling. *Sci. Rep.* **6**, 35156; doi: 10.1038/srep35156 (2016).

## Figures and Tables

**Table 1:** Curie temperature (*T*_*C*_), change in magnetic entropy (Δ*S*_*M*_), relative cooling power (RCP) and cost for selected magnetocaloric materials.

Nominal Composition	*T*_*C*_ (K)	∆S_*M*_ (J-kg^−1^K^−1^) (μ_o_*H* = 5T)	RCP (J-kg^−1^) (μ_o_*H* = 5T)	Cost per 100 gm ($)	Ref.
(Fe_70_Ni_30_)_99_Cr_1_	398	1.58	548	7.6	This work
(Fe_70_Ni_30_)_97_Cr_3_	323	1.49	436	8.1	This work
(Fe_70_Ni_30_)_95_Cr_5_	258	1.45	406	8.6	This work
(Fe_70_Ni_30_)_94_Cr_6_	245	1.22	366	8.8	This work
(Fe_70_Ni_30_)_93_Cr_7_	215	1.11	306	9.1	This work
NiFeCoCrPd_0.25_ (as rolled/annealed)	~210	0.9/0.82	170/150	1526	17
NiFeCoCrPd_0.50_ (as rolled/annealed)	~290	0.87/0.83	—	2984	17
(Fe_70_Ni_30_)_95_Mn_5_	338	1.45	470	7.3	31
(Fe_70_Ni_30_)_92_Mn_8_	340	1.67	466	7.3	5
(Fe_70_Ni_30_)_89_ Zr_7_B_4_	353	2.8	330	62.1	18
(Fe_70_Ni_30_)_89_B_11_	381	2.1	640	129	13
(Fe_70_Ni_30_)_96_Mo_4_	300	1.67	432	8.3	21
Gd	295	7.2	~400	450	49
Gd_5_Ge_1.9_Si_2_Fe_0.1_	300	7.1	630	409.4	56

The cost of the materials was calculated using pure element cost.
